# Assessment of dementia knowledge and its associated factors among final year medical undergraduates in selected universities across Malaysia

**DOI:** 10.1186/s12877-022-03148-7

**Published:** 2022-05-24

**Authors:** Chee Mun Chan, Marjorie Jia Yi Ong, Adam Aiman Zakaria, Monikha Maria Visusasam, Mohd Fairuz Ali, Teh Rohaila Jamil, Azimatun Noor Aizuddin, Aznida Firzah Abdul Aziz

**Affiliations:** 1 Department of Family Medicine, Faculty of Medicine, Universiti Kebangsaan Malaysia, Jalan Yaacob Latif, Bandar Tun Razak, 56000 Cheras, Kuala Lumpur, Malaysia; 2 Class of 2021/2022, Faculty of Medicine, Universiti Kebangsaan Malaysia, Jalan Yaacob Latif, Bandar Tun Razak, 56000 Cheras, Kuala Lumpur, Malaysia; 3 Department of Community Health, Faculty of Medicine, Universiti Kebangsaan Malaysia, Jalan Yaacob Latif, Bandar Tun Razak, 56000 Cheras, Kuala Lumpur, Malaysia

**Keywords:** Dementia, Geriatric, Medical education, Dementia knowledge

## Abstract

**Background:**

The elderly population in Malaysia are projected to reach almost one third of the total population by 2040. The absence of a National Dementia Strategy (NDS) in preparing the healthcare services for the ageing population is compounded by the lack of assessment of preparedness of future healthcare workers to manage complications related to ageing i.e., dementia. Studies in countries with NDS demonstrated lack of dementia knowledge among medical undergraduates. Hence, this study aimed to assess the knowledge on dementia among final year medical undergraduates in Malaysia and its associated factors, using the Dementia Knowledge Assessment Scale (DKAS).

**Methods:**

This cross-sectional study, employed multistage sampling method to recruit final year medical undergraduates from eleven selected public and private medical institutions across Malaysia. Online self-administered measures were delivered to final year medical undergraduates through representatives of medical students’ society after approval from Deanery and institutional ethics board of participating universities. The measure collected demographic information, previous dementia exposure (i.e., formal or informal) and the 25-item Likert scale DKAS. Bivariate analysis and linear regression were conducted to confirm factors influencing dementia knowledge components.

**Results:**

A total of 464 respondents from 7 universities participated in this study. Overall dementia knowledge among respondents with and without exposure, was low, with average score of 29.60 ± 6.97 and 28.22 ± 6.98, respectively. DKAS subscales analysis revealed respondents scored highest in care consideration subscale (9.49 ± 2.37) and lowest in communication and behaviour subscale (4.38 ± 2.39). However, only causes and characteristic subscale recorded significantly higher knowledge score among respondents with previous exposure (7.88 ± 2.58) (*p* =0.015). Higher knowledge of dementia was associated with previous formal dementia education (*p*=0.037) and informal occupational/working experience in caring for dementia patients (*p* = 0.001). Informal occupational/working experience (B = 4.141, 95% CI 1.748–6.535, *p* = 0.001) had greater effect than formal education (i.e. lectures/workshops) (B = 1.393, 95% CI 0.086–2.700, *p* = 0.037) to influence respondents’ knowledge on dementia.

**Conclusion:**

Dementia knowledge among final year medical undergraduates is low. To improve dementia knowledge, Malaysian medical curriculum should be reviewed to incorporate formal education and informal occupational/working experience, as early as in undergraduate training to help prepare future healthcare providers to recognise dementia among ageing Malaysians.

## Background

Rapid demographic shifts with increase in life expectancy and reduction in birth rates have resulted in higher prevalence of ageing population. According to the 2015 World Report on Ageing and Health by World Health Organisation (WHO), it was highlighted that the number of individuals aged 60 years and above is expected to increase over the next 15 years and double by 2050, especially in middle-income countries in the Asia-Pacific region [1].

Dementia is one of the significant public health issues that is associated with ageing population, which is always described as a neurocognitive brain disorder that affects thinking, memory, behaviour, and emotion. The most common cause of dementia is Alzheimer’s disease while others include vascular disease, dementia with Lewy bodies and frontal-temporal dementia [2]. According to the latest report by Alzheimer Disease International (2020), it is currently estimated that over 50 million people worldwide are living with dementia and this trend is expected to rise steadily to 152 million by 2050, especially in low and middle-income countries. This is also similar to a new case of dementia in the world occurring every 3 seconds. High prevalence of dementia gives rise to burdening medical costs, especially for developing countries with an average of USD818 billion worldwide annually [2].

In Malaysia, a study in 2015 reported that the estimated prevalence of dementia was 123,000 people. It is expected to increase from 261,000 in 2030 to 590,000 by 2050 [3]. This indirectly indicates that our population is aging, as supported by the National Health and Morbidity Survey 2019 (NHMS 2019) which demonstrated that 16.3% of study population were aged 60 and above and this figure is expected to rise by 15% in 2040 [4]. Thus, healthcare services should be prepared to cater to the needs of the aging population which includes provision of dementia-related care services.

Many family caregivers and patients depend on their primary care physicians and family doctors for advice in anticipation of disease progression, but also physical and mental preparation of how to manage the changing conditions associated with the disease [5]. This three-way relationship is commonly known as the dementia care triad. However, the literature suggests that the incompetency of healthcare providers in making early diagnosis of dementia have resulted in poor outcomes for both patients and caregivers. Early recognition of dementia symptoms provides explanation to the patients and put an end to their suspicions. Subsequently being forewarned, patients who are in the early stages can plan ahead in terms of their own legal, financial and future support options and treatment while they still have the capacity and make these wishes known to family members [6]. Studies have also shown that undetected dementia in younger individuals may be associated with work disability and be released from their job. As a result of losing employment at young age, they will experience significant financial problems not only to themselves, but also to family members [7]. On the other hand, caregivers received inadequate information on the care of the disease, misinterpretation of behaviours and increased caregiver stress due to failure to receive appropriate support. Many studies cited that this is due to poor level of knowledge and negative attitude towards dementia patients among healthcare providers [5, 8].

The major contributing factor towards poor attitude of healthcare providers is the stigma towards dementia [9]. Stigma is defined as negative beliefs and attitudes, which are often discriminatory towards a specific group of individuals. The literature suggests that the adverse relationship of stigma towards dementia outcome as it impedes proper patient care [8]. Unfortunately, a literature review in 2017 cited that research that specifically targeted dementia-related stigma for the past decade were limited. Most of the studies were conducted in United States and Europe while some countries, particularly in Asia only have single publication [10]. To further understand dementia as a stigma, a conceptualization is needed which sufficiently expanded on various aspects such as the nature of attribute, the social process, contributing factors towards the stigma and the experience of the individuals involved [11]. For instance, the misconception of memory loss in dementia as a natural aging process has led to negative association to the disease. Among healthcare providers, the lack of reciprocity in patients contributes to the development of stigma towards dementia. General reciprocity can be defined as recognizing helpful and beneficial action and responding in reciprocal manner. To elaborate, primary caregivers often feel a lack of ‘meaningful experience’ and ‘decreased sense of sincere social contact’ demonstrated by dementia patients. As a result, physicians may be hesitant to officially diagnose a patient with dementia concerning the emotional effect, and studies have shown that 60% of elderly remain undiagnosed. Besides, studies have also shown that feeling of helplessness, concerning the emotional effect of patients, was frequently reported among general practitioners and it was more prevalent in those who had not undergone any education about dementia. These stigmas will become obstacles in early screening of dementia and many attributed them to the lacking of knowledge regarding dementia [12]. As the disease advances, it can be detrimental for the patient who is at an early stage of dementia, as they are more likely to engage in unsafe activities of daily living i.e., cooking, driving and finance management, which can be dangerous to patients who have not received a formal diagnosis. Their safety is not appropriately addressed, and they are at risk of succumbing to injuries or potentially vulnerable for financial abuse or theft. As a result, this emphasises the significance of early dementia diagnosis to provide the best prognosis for patients and caregivers.

In recognition of the impact of increased stigma of dementia towards patients and families, many countries have developed and implemented National Dementia Strategies (NDS) in response to the increasing prevalence of dementia [13]. As an initiative to reduce dementia-related stigma and enhanced care quality to dementia patients, the United States developed the National Plan to Address Alzheimer’s Disease in 2018 which aimed to educate healthcare providers about the recent research findings, clinical tools for assessment, diagnosis and management of cognitive impairment. The preparedness of healthcare services is imperative to provide accurate information to dementia patients and caregivers including benefits of early diagnosis and methods to cope with the physical, cognitive, emotional and behavioural symptoms of disease. Early diagnosis allows time to plan and prepare for future as mentioned above, leading to more positive outcome for both patients and caregivers [14]. This highlighted the importance to empower geriatric education and knowledge in healthcare providers, citing another US-based study whereby it was reported that final year medical undergraduates who were more exposed to geriatric education in their medical curriculum had better knowledge than first year medical undergraduates [15].

In South East Asia, Gerald and colleagues conducted a comparative study between the effectiveness of old and new medical curriculum, among Singapore medical undergraduates. The new curriculum incorporated geriatric education as early as year 2 of the medical curriculum, compared to the old curriculum which initiated geriatrics only in year 5 [16]. They reported that year 2 and year 5 medical undergraduates in the new cohort have statistically significant higher mean scores in geriatric knowledge compared to the old curriculum cohort. Thus, this concluded that the newly introduced longitudinal interprofessional geriatric medicine curriculum had significantly improved geriatric knowledge among medical undergraduates in Singapore. This proves that dementia education is crucial and should be incorporated into the national medical curriculum to ensure that future generations are better prepared and participate in management of dementia [17].

Over the years, many studies have found that healthcare students are inadequately trained and educated on dementia [18–21]. To date, there is a lack of data about the evaluation of knowledge of dementia among medical undergraduates to gauge the impact on future dementia management in Malaysia. Hence, this study aimed to assess the knowledge on dementia among final year medical undergraduates in Malaysia, who will contribute as future healthcare providers using the Dementia Knowledge Assessment Scale (DKAS) [22].

## Methods

### Sampling

This was a cross-sectional study conducted on final year medical undergraduates recruited from seven Malaysian medical institutions during May – August 2021 period. A total of thirty-two public and private medical schools were approached to participate in this study, out of which only eleven universities provided preliminary agreement to participate in this study. However, Deanery approval was only granted by seven universities. The total number of final year medical undergraduates for the 2020/2021 academic session of each university, who had completed at least two years of clinical training, was obtained. Using the formula for finite population sampling, 95% confidence interval from a sampling frame of 825, with 75% attrition rate for failure to respond to online survey, 464 samples were required.

### Data collection

**Fig. 1:**
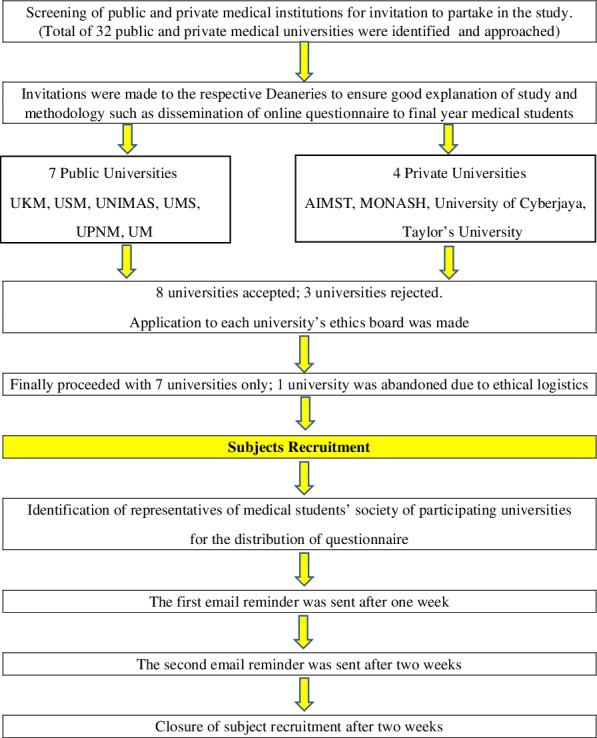
Study flow chart

The measures were distributed via the representatives of medical students’ society of participating universities after ethical board approval were obtained from each participating institution. The participants accessed the self-administered virtual measures via QR codes sent to their emails or mobile smartphones. Each respondent was allowed only one attempt. The online data gathering system ensured that all items on the measures had to be answered before respondents could proceed to the subsequent questions, hence ensuring no incomplete surveys were submitted. If duplicate entries were recorded from same respondent, only the first response with an earlier timestamp will be considered for analysis. No post-measure editing was allowed once submitted. Respondents were advised to complete the measure within 5 minutes to avoid loss of internet connection and to avoid having to ‘refresh’ the measure page (Fig. 1).

### Instrument

An online measure was developed on Microsoft Forms portal. The measure consisted of two sections. The first section, gathered the respondents’ demographic information included age, gender, name of university, and previous dementia exposure. Previous dementia exposure was assessed with the following questions: previous exposure to formal dementia education (such as attended educational session for health professionals OR training course / workshop about dementia), if there was any immediate family member (parents, siblings, spouse, children, anyone under your guardianship) diagnosed with dementia, and if there was any direct occupational/working experience in caring for dementia patients. The second section was the Dementia Knowledge Assessment Scale (DKAS).

The DKAS is a Likert scale that was originally developed as a 27-item knowledge questionnaire in the Wicking Dementia Research and Education Centre, University of Tasmania, Australia in 2015, with correct and incorrect statements about dementia [23]. It was revised in 2017 into a 25-item Likert scale which was the version  used in this study. The DKAS was chosen as it exhibited good validity and reliability [24, 25]. The DKAS covers four subscales on dementia: Causes and Characteristics, Communication and Behaviour, Care Considerations, and Risks and Health Promotion. The original English version of DKAS was used for this study. The questionnaire has a grand total score of 50 marks, with the following Likert scale scoring system: score 2 points for an answer of ‘true’ to a truthful statement or, ‘false’ to an untrue statement; score 1 point for an answer of ‘probably true’ to a truthful statement or, ‘probably false’ to an untrue statement; and score 0 points for an answer of ‘true’ or ‘probably true’ to an untrue statement, ‘false’ or ‘probably false’ to a truthful statement, or for an answer of ‘I don’t know’. Permission to use the questionnaire was granted by the authors from University of Tasmania. The questionnaire was piloted on a sample of 35 fourth year medical undergraduates to test for its technical usability and stability prior to distribution. No changes were made after the pilot test.

### Statistical analyses

Data analysis of this study was performed using the Statistical Package for Social Science (SPSS) version 25. Categorical data was expressed as frequencies and percentages, while continuous data was expressed in means and standard deviations. A comparison of dementia knowledge between two groups of respondents (with and without dementia exposure) was performed with independent sample t tests. The differences in results were analysed in several factors such as DKAS subscales (Causes and Characteristics, Communication and Behaviour, Care Considerations, Risks and Health Promotion) as well as previous dementia exposure (formal education exposure, family members with dementia and informal occupational/working experience in caring for dementia patients). Multiple linear regression was used to predict significant factors of previous dementia exposure associated with the total score of DKAS. A *P* value of less than 0.05 was considered statistically significant.

## Results

### Data characteristic and demographic information

Total of 464 out of the 826 students recruited completed the measure, making the response rate of 56.2%. The average completion time for the survey was recorded at 10 minutes per respondent. 70.9% of the students were females and the mean age was 24.19 ± 0.62 years. UKM had the highest number of respondents (29.96%) while UPNM recorded the lowest (7.11%). Our results also demonstrated only 50.4% of the respondents had previous dementia exposure. The detailed characteristics of the study population is summarised in Table 1.

**Table 1:** Characteristics of Study Respondents (*N* = 464)

Indicator	Mean ± SD	N (%)
**Age (years)**	24.19 ± 0.62	
**Gender:**		
Female		329 (70.9)
Male		135 (29.1)
**Place of study:**		
Universiti Kebangsaan Malaysia (UKM)		139 (29.96)
Universiti Sains Malaysia (USM)		49 (10.56)
Universiti Putra Malaysia (UPM)		57 (12.28)
Universiti Malaysia Sarawak (UNIMAS)		87 (18.75)
Universiti Pertahanan Nasional Malaysia (UPNM)		33 (7.11)
University of Cyberjaya (UOC)		48 (10.34)
Others		51 (10.99)
**Previous Dementia Exposure:**		
Yes		234 (50.4)
No		230 (49.6)
**Previous Formal Dementia Education Exposure** **(Lectures/Workshops)**		
Yes		180 (38.8)
No		284 (61.2)
**Previous Informal Dementia Exposure** **(Family members):**		
Yes		34 (7.3)
No		434 (92.7)
**Previous Informal Dementia Exposure** **(Occupational/working experience in caring for dementia patients):**		
Yes		35 (7.5)
No		429 (92.5)

### Comparison of dementia knowledge between groups

We further stratified our respondents into two groups; with previous dementia exposure and without previous dementia exposure. Independent t test was used to compare the dementia knowledge between these two groups of respondents from various medical universities (Table 2). We found that students with exposure scored statistically significant higher mean score (29.60 ± 6.97) than those without exposure (*P* < 0.034). This trend was seen across five universities, especially USM and UPM which have significantly higher mean score of 31.25 ± 6.54 and 31.24 ± 6.47, respectively than respondents without previous exposure (*P* < 0.05). However, only UNIMAS, with 60.92% of respondents with previous exposure scored lower (28.02 ± 7.15) than those without exposure (29.71 ± 6.17). Respondents labelled as “others” were not included in this analysis as they comprised of only small percentage of the total batch cohort, thus rendered insufficient to represent the university for knowledge scores comparison.

**Table 2:** Differences of DKAS scores between previous dementia exposure and universities

Characteristics	N (%)		Mean ± SD	***P*** value^**a**^
**Total Population**	Yes	234 (50.4)	29.60 ± 6.97	0.034*
	No	230 (49.6)	28.22 ± 6.98	
**University**				
Universiti Kebangsaan Malaysia (UKM)	Yes	63 (45.32)	30.08 ± 6.54	0.226
	No	76 (54.68)	28.54 ± 8.09	
Universiti Sains Malaysia (USM)	Yes	24 (48.98)	31.25 ± 6.54	0.02*
	No	25 (51.02)	27.08 ± 5.54	
Universiti Putra Malaysia (UPM)	Yes	33 (57.89)	31.24 ± 6.47	0.05*
	No	24 (42.11)	27.71 ± 6.71	
Universiti Malaysia Sarawak (UNIMAS)	Yes	53 (60.92)	28.02 ± 7.15	0.261
	No	34 (39.08)	29.71 ± 6.17	
Universiti Pertahanan Nasional Malaysia (UPNM)	Yes	14 (42.42)	29.14 ± 5.46	0.258
	No	19 (57.58)	26.58 ± 6.87	
University of Cyberjaya (UOC)	Yes	25 (49.02)	29.88 ± 6.99	0.608
	No	26 (50.98)	28.92 ± 6.33	

^a^ indicates results from independent t test; *significance level at *P* < 0.05

### DKAS subscales analysis between groups

We compared the mean score of subscales between respondents with and without previous dementia exposure. Table 3 depicted respondents with previous exposure scored higher mean score in all four subscales than those without, with highest score in care consideration subscale (9.49 ± 2.37) and lowest in communication and behaviour (4.38 ± 2.39). It was also reported that only the first subscale (causes and characteristics) recorded significant difference in the mean score between the two groups (*P* < 0.015).

**Table 3:** Scores of Subscales of DKAS with previous dementia exposure stratification

Subscales	#Items (Total score)	Have Previous Dementia Exposure, ***N*** = 234 (Mean ± SD)	No Previous Dementia Exposure, ***N*** = 230 (Mean ± SD)	***P*** value^**a**^
a) Causes and Characteristics	7 (14)	7.88 ± 2.58	7.30 ± 2.5	0.015*
b) Communication and Behaviour	6 (12)	4.38 ± 2.39	4.11 ± 2.45	0.243
c) Care Considerations	6 (12)	9.49 ± 2.37	9.27 ± 2.47	0.333
d) Risks and Health Promotion	6 (12)	7.85 ± 2.60	7.53 ± 2.65	0.185

^a^ indicates results from independent t test; *significance level at *P* < 0.05

### Predicting factors of previous dementia exposure affecting dementia knowledge

As shown in Table 4, respondents with previous formal or informal dementia exposure scored higher than those without any exposure. Respondents with occupational/working experience in caring for dementia patients have the highest mean score of 32.74 ± 7.07 while the lowest mean score was seen in respondents with formal dementia education exposure, 29.77 ± 6.94. Multiple linear regression analysis showed that formal education and informal occupational/working experience in caring for dementia patients were significant predictors of DKAS (*P* < 0.05). It was predicted that respondents with previous formal education and informal occupational/working experience in caring for dementia patients scored 1.4 and 4.1 points higher respectively, on DKAS compared with respondents without such experience.

**Table 4:** Factors of previous dementia exposure affecting DKAS score

Factors	Mean ± SD^**a**^	B-value	95% CI	***P*** value^**b**^
Previous formal dementia education (workshops/lectures)				
Yes	29.77 ± 6.94	1.393	0.086–2.700	0.037*
No	28.37 ± 7.00			
Previous Informal Dementia Exposure (family members)				
Yes	30.29 ± 8.99	1.489	−0.962-3.941	0.233
No	28.80 ± 6.83			
Previous Informal Dementia Exposure (occupational/working experience in caring for dementia patients)				
Yes	32.74 ± 7.07	4.141	1.748–6.535	0.001*
No	28.60 ± 6.92			

^a^ indicates results from independent t test; ^b^ indicates results from multiple linear regression;

*Significance level at < 0.05

## Discussion

The primary objective of this study was to establish the baseline level of dementia knowledge among final year medical undergraduates in Malaysia using DKAS. Our study revealed respondents with and without previous exposure had low knowledge of dementia with mean scores of 29.60 ± 6.97 and 28.22 ± 6.98, respectively. Comparatively by using DKAS, our respondents scored much lower than Australian healthcare students who recorded a mean score of 34.48 ± 8.30 among 173 students [26]. Similarly, Fahad and his co-workers who scored DKAS differently, based on a maximum score of 25, reported that healthcare students from two departments in Kuwait University had low knowledge of dementia of 15.87 ± 3.33 and 16.02 ± 2.82, which are much higher scores compared to Malaysian medical undergraduates in our study [27].

Our findings also indicated that there are discrepancies of dementia exposure in all medical institutions, as the percentage of respondents with previous exposure was similar to those without exposure. Students with previous exposure have higher level of knowledge of dementia. This finding is analogous to many studies, such as the study among Korean nursing students where respondents with relevant educational training experience scored significantly higher than those without [28]. Regionally in Malaysia, a local study by Devinder and colleagues assessed 786 health science students from UKM also reported greater students’ knowledge of aging among those who had taken geriatric courses or modules [29]. Our findings also reinforced suggestions by Panmial and colleagues who highlighted significantly higher knowledge of dementia among UPM medical and nursing students with previous exposure [30].

Based on the results of the four subscales of DKAS, our respondents scored highest in the third subscale (Care Considerations), replicating the findings by Fahad and colleagues. Malaysia is an Islamic-dominant religion country similar to Kuwait, thus caring for elders is important as advocated by the religion. It is important for elderlies to live with their families, to spend time with their children for an improved quality of life and well-being compared to living in nursing homes [27]. Similar values, culture and religious norms towards older adults are also held by the multicultural society of Malaysia, resulting in a more positive attitude in caring for elderly [29]. On the other hand, our respondents scored lowest in the second subscale (Communication and Behaviour). This matter can be viewed from two perspectives; the changes in mode of medical education due to the COVID-19 pandemic and the patients’ dementia progression. The pandemic has resulted in reduced clinical exposure and cancellation of face-to-face clinical sessions. This combination was cited to have impacted delivery methods and quality of medical education, particularly on final year medical undergraduates who are expected to acquire certain competencies and skills before starting their career [31].

Furthermore, communication deficits, such as loss of verbal fluency and concentration are also commonly experienced by dementia patients, and lack of experiential learning to handle these signs and symptoms renders the medical undergraduates at a disadvantage. Although the communication-related questions structured in DKAS showed no direct correlation to negative attitudes in caring for dementia patients, we postulate that certain communication barriers identified compounded existing inherent stigmatization and misconceptions. A study conducted by Theresa and colleagues in Australia explained that age-related stereotypes are the norm, such as poorer medical diagnosis, often helpless and will eventually become senile, will have unfulfilling life later, thus do not deserve healthcare services. The authors concluded that this observation was possibly attributed by lack of communication and insufficient exposure to understand the older population. As a result, this will lead to discriminatory practices and preferential bias to focus on younger patients. Therefore, it is important to address the communication deficit leading to stigmatization and have enhanced communication skills to ensure effective communication and care provision, especially in the pandemic era [32]. However, a literature review by Ledia and co-workers cited that although many intervention programmes were conducted to improve students’ attitude and knowledge, surprisingly none specifically addressed the need to improve the communication skills. To date, it is not possible to suggest an optimal length of exposure to enhance communication skills, even though many studies reported positive results associated with previous exposure to dementia patients. As a result, direct contact or practical immersion without adequate preparation can incur feelings of inhibition and intimidation among medical undergraduates when interacting with dementia elders [32]. In addition to that, it is also worth emphasising that students with previous exposure have significantly higher mean scores than those without exposure in the first DKAS subscale (Causes and Characteristics). We postulated that they were more educated theoretically about the pathological aspects of the brain regarding dementia [32]. Another plausible reason for the significant differences could be the increased teaching hours and coverage of dementia topics which may have improved the knowledge of dementia among the students [33].

Given the substantial evidence supporting the importance of dementia exposure, our second objective of this study was to determine significant predictors of previous exposure to increased dementia knowledge. Firstly, our study highlighted that previous formal dementia education exposure such as lectures and workshops in the medical curriculum was associated with increase in dementia knowledge. A review by  Basri and colleagues supported our findings as they cited specific curricular modules on dementia that focused on understanding dementia theoretically and were vital in improving students’ knowledge in dementia. Previous studies indicated that the modules should target on causes, prevention methods, risk factors and treatments to bridge the gaps in understanding dementia [34].

The effectiveness of integrated dementia education was also seen in among Norwegian undergraduate nursing students who were found to have significantly higher knowledge than Maltese nursing students [33]. The improvement of knowledge was due to the introduction of the 2015 National Dementia Plan in Norway, which focused on the need for increased dementia-related knowledge and skills among healthcare professionals. The addition of dementia topics in undergraduate healthcare students’ curriculum and increased exposure to dementia patients via clinical placements to produce competent and qualified healthcare personnel in dementia [35]. In contradistinction to countries with implementation of national dementia strategy, a study in China  indicated that approximately 70% of year 3 medical undergraduates had no knowledge regarding elderly issues. Liu and his colleagues hypothesised that this was due to the limited compulsory gerontology education in China [36]. Similarly, for Malaysia, with its delayed implementation of a National Dementia Strategy, our study findings have proven that efforts to include gerontology courses and enhanced hands-on experience with dementia patients is urgently needed.

Our study also reported that informal occupational/working experience in caring for dementia patients was significantly associated with increased knowledge of dementia. Comparative studies have shown that medical undergraduates exposed to clinical hands-on experience with patients with dementia have better knowledge and attitude compared to those who only completed online modules. It is important to integrate clinical exposure in the curricular modules as this will boost students’ confidence, comfort and understanding on how to approach and interact with patients. At the same time, they will also learn the humanistic side of the disease which later synapses the scientific findings and practical experience to encourage deeper understanding on dementia [34]. Similarly, The Boston University’s Partnering in Alzheimer’s Instruction Research Study (PAIRS) programme, aimed at enhancing geriatric healthcare issues in medical education had successfully shown improvements in dementia knowledge among 79 first year medical undergraduates. The study highlighted that interaction with Alzheimer’s patients enhanced student awareness of care and support-related issues encountered by patients and their families. Through clinical exposure, students were exposed to psychological challenges of the chronic disease and the impact to the caregivers and family system resulting in increased students’ awareness and compassion to care for elders with dementia [37].

## Strength and limitation

This is the first nationwide study to establish the baseline dementia knowledge among the largest sampled study of medical undergraduates in final year from various medical institutions. Our online survey platform was useful for yielding no apparent nonsense entry through forced choice designed answers. However, several limitations were identified in this study. Firstly, our respondents were recruited from selected public and private universities who agreed to participate, thus undermining the generalisation to the population of Malaysian medical undergraduates. Higher levels of recruitment are required, to elucidate the potential differences in institutions’ geriatric curriculum, impact of the curriculum to improve knowledge of dementia among the medical undergraduates. Hence, a better association can be highlighted to reflect on the importance of previous dementia exposure. Secondly, respondents may have accessed materials on internet searching for ‘correct answers’ as they were not monitored during the survey. Therefore, bias results may occur among some respondents who have obtained a high mean score of DKAS. Lastly, we were unable to verify self-reported previous exposure by our respondents. Detailed data regarding previous formal and informal dementia such as duration and frequency of exposure per week exposure were not collected.

## Future implication

Given the poor knowledge of our current budding medical graduates, it is important to address that a National Dementia Strategy should be outlined in Malaysia as this will support the development of dementia education aimed to improve institutional care, treatment practices and enhance the public’s awareness towards the spreading non-communicable burden of dementia. Future research should explore the attitudes and perceptions of undergraduate medical undergraduates towards dementia patients as this is a principal factor to enhance their approach in geriatric care.

## Conclusion

In conclusion, dementia knowledge among final year medical undergraduates is low. To improve dementia knowledge, Malaysian medical curriculum should be reviewed by incorporating formal education and informal occupational/working experience, as early as in undergraduate training to help prepare future healthcare providers to recognise dementia among ageing Malaysians.

## Data Availability

The datasets generated and/or analysed during the current study are not publicly available but are available from the corresponding author on reasonable request. Permission to share datasets publicly was not obtained from some participating universities.
